# Determinants of cholera fatalities in Malawi: A case-control study of patient-level and clinical management factors in the 2022–23 outbreak

**DOI:** 10.1371/journal.pgph.0004335

**Published:** 2025-10-09

**Authors:** Ronald Chitatanga, Alex Thawani, Hope Chadwala, Amon Chirwa, Collins Mitambo

**Affiliations:** 1 Antimicrobial Resistance National Coordinating Centre, Public Health Institute of Malawi, Lilongwe, Malawi; 2 University of North Carolina Project-Malawi, Lilongwe, Malawi; 3 Research Department, Public Health Institute of Malawi, Lilongwe, Malawi; 4 Kamuzu Central Hospital, Lilongwe, Malawi; PLOS: Public Library of Science, UNITED STATES OF AMERICA

## Abstract

Malawi experienced its deadliest cholera outbreak in 2022, reporting over 50,000 cases and more than 1,700 deaths. This situation was further exacerbated by the Tropical Cyclone Freddy, which caused widespread damage to health infrastructure and strained Malawi’s limited healthcare resources. Despite the scale of the outbreak, no evaluations were conducted to identify risk factors associated with cholera-related mortality among hospitalized individuals. This study filled that gap by characterizing the clinical and treatment-related factors contributing to cholera mortality in Malawi. A retrospective matched case-control study was conducted in August 2023 across four high-burden cholera districts: Blantyre, Mangochi, Lilongwe, and Nkhatabay. Medical records of 174 laboratory-confirmed cholera patients admitted between March 2022 and September 2023 were reviewed, including 87 matched pairs of deceased (cases) and surviving (controls) patients by age group and district. Data were extracted using a standardized mortality audit tool capturing socio-demographic characteristics, care-seeking behaviour, clinical status, and treatment received. Conditional logistic regression was used to identify factors associated with cholera mortality. Inadequate intravenous fluid management within the first 6 hours of admission was the strongest predictor of mortality (adjusted OR = 45.26, 95% CI: 9.09–225.30, p < 0.001). Other factors such as clinical status on admission showed a trend toward association but did not reach statistical significance. Timely and appropriate intravenous fluid administration within the first 6 hours of care is critical to reducing cholera mortality. We highlight an urgent need to strengthen the early response capacity in cholera treatment units, particularly during climate-related public health emergencies.

## Introduction

Between 1990 and 2019, cholera was responsible for an estimated three million global deaths [1]. These fatalities ranged from 83,045 in 1990–117,167 in 2019, demonstrating an overall increasing trend in mortality [1]. Alarmingly, the African Region accounted for the highest number of cholera-related deaths in 2019, comprising 79% of the global total [1]. Within Africa, the Central African Republic and Nigeria reported the highest mortality rates, at 44.63 and 40.74 per 100,000 of the population, respectively [1]. This concerning rise in cholera mortality has persisted despite global advancements in case management, hygiene, and sanitation, underscoring the enduring challenge cholera poses in global health.

Cholera incidence has been closely linked to climate change. Variations in rainfall patterns, temperature, and the occurrence of floods and droughts have been associated with increased cholera incidence in both African and Asian countries [2]. Heavy rainfall can lead to flooding, causing contamination of rivers through the mixing of water with sewer systems. Conversely, low rainfall during droughts reduces access to sufficient safe water, thereby heightening the risk of cholera outbreaks [2]. In Africa, countries such as Ghana, Tanzania, and Zambia have demonstrated a positive correlation between rainfall and cholera epidemics [2].

In Malawi, cholera was first reported in 1973 during the seventh cholera pandemic wave caused by *Vibrio cholerae* serotype Inaba, which affected East Africa [3]. Since then, cholera has remained endemic in Malawi, with seasonal outbreaks leading to as many as 22,427 cases in a single year (1975) [3]. Despite its endemic status, Malawi experienced its deadliest cholera outbreak beginning in March 2022 [4]. Where as of March 6, 2023, this outbreak resulted in 51,568 reported cases and 1,612 fatalities (Case Fatality Rate = 3.1%), affecting all districts in the country. Similar to outbreaks in other African nations, this crisis was triggered and exacerbated by severe rainfall events, including Tropical Cyclones Ana, Gombe, and Freddy [3]. These cyclones displaced communities and destroyed more than 80 healthcare facilities, further compounding the health crisis [3–5].

Despite systemic improvements in Malawi’s healthcare infrastructure, including experience gained from managing outbreaks of COVID-19 in 2020 and wild poliovirus type 1 in 2022 [6,7], the 2022 cholera outbreak was the worst in the country’s history. This outbreak recorded a case fatality rate of 3.1%, which is more than the 1% threshold by the WHO [8]. However, by the start of the outbreak, Malawi had already implemented several preventive measures, including the introduction of the Oral Cholera Vaccine (OCV) beginning in June 2017 [9] and community-wide cholera awareness campaigns. These interventions, alongside improvements in water, sanitation, and hygiene (WASH) services, were expected to mitigate cholera transmission and severity. Yet, the outbreak’s unprecedented impact raises critical questions about the factors contributing to its severity, particularly concerning clinical management.

Since the onset of the cholera outbreak in March 2022, no updated evaluations of patient-level risk factors influencing mortality among hospitalized cholera patients had been conducted. This study addressed this gap by characterizing the clinical and treatment-related factors prevalent in fatal cholera cases and investigating the relationship between these factors and mortality outcomes.

## Methodology

### Design

This was a retrospective review of patient medical records belonging to cholera patients admitted between 1 March 2022 to 30 September 2023. Files from treatment units located in the District Health Offices of Nkhatabay, Lilongwe, Mangochi and Blantyre districts were enrolled. A case-control analytic approach was applied, where deceased cholera patients (cases) were matched with controls, who were patients also treated for cholera and discharged from the same treatment unit. Data collection took place between 21 August - 1 September 2023.

### Study setting and eligibility criteria

The study was conducted in four cholera high-burden districts across Malawi’s three regions, selected based on national cholera surveillance reports from the Malawi Ministry of Health [10]. As of 14 March 2023—one year into the outbreak—Lilongwe (Central Region) had reported 11,219 cases and 532 deaths, Mangochi (South-Eastern Region) 8,350 cases and 123 deaths, Blantyre (Southern Region) 7,577 cases and 202 deaths, and Nkhatabay (Northern Region) 1,517 cases and 44 deaths [10]. These districts were purposefully selected to reflect geographic diversity and disease burden. The study included medical records of all patients admitted to cholera treatment units in these districts with a confirmed cholera diagnosis. Eligibility was limited to patient-files with documented laboratory-confirmed cholera, either through a positive rapid diagnostic test or a positive cholera stool culture. Patient files of both deceased and discharged patients were eligible for enrolment.

### Survey Tool

A standardised mortality audit tool was developed and used to retrospectively extract patient medical records in cholera units across the selected districts. The tool was structured around four main domains:

1. **Socio-demographic characteristics**: This section captured information on patient sex (M, F), age group (<10, 10–19, 20–29, 30–39, 40–49, 50–59, ≥ 60), district of diagnosis (Nkhatabay, Lilongwe, Mangochi, Blantyre), and marital status (single, married, unknown).
2. **Identified health-seeking behaviors**: This section assessed patient behaviour prior to hospital admission, specifically: Time to hospital presentation (categorized as <6 hours, 6–24 hours, 24–48 hours, > 48 hours) after symptom onset and cholera vaccination status (vaccinated, unvaccinated, unknown).
3. **Risk factors**: Comorbidities such as Human Immunodeficiency Virus (HIV) infection (positive, negative, unknown).
4. **Patient treatment assessment**: This section focused on the clinical condition on admission (as per hospital assessment by the nurse or clinician) and treatment received during hospitalisation. It included: clinical status on admission (critical vs. stable as per vital sign assessment), the initial hydration status on admission (categorized as no dehydration, some dehydration, or severe dehydration, in accordance with the WHO classification scale for dehydration [11], which is based on clinical signs such as skin turgor, level of consciousness, and capillary refill), adequacy of fluid management (assessed based on whether the type, volume and timing of intravenous fluids administered within the first 6 hours aligned with the standard cholera treatment guidelines for managing dehydration), antibiotic treatment (whether antibiotics were given or not), and time spent in the ward before discharge or death (categorized as <6 hours, 6–24 hours, 24–48 hours, 48 hours–7 days, and >7 days).

### Data collection

Data from the medical files were collected by trained research Medical Officers using the mortality audit tool which was designed and deployed through Kobo Toolbox, an open-source data collection, analysis and management tool [12]. All patient information was treated confidentially and securely stored on a password-protected computer accessible only to the study team.

### Sample size estimation and sampling technique

A total of 174 cholera medical files were enrolled in this study, with 50% classified as cases (patients who died) and 50% as controls (survivors), maintaining a 1:1 ratio. The sample size was determined to achieve a power of 99.97%, assuming a binomial distribution and applying an arcsine transformation to standardize the effect size (h = 0.81). The power analysis was conducted with a significance level of 0.05 under a two-sided alternative hypothesis, ensuring that the study was adequately powered to detect differences between the two groups.

All fatal cholera patients (cases) were initially identified in each cholera unit and then a random sampling technique was applied for selecting controls. These controls were selected systematically, where every second patient file was chosen for enrolment into the study.

#### Controls.

Each control was matched to a case based on age-range and the district of cholera diagnosis. The age ranges were: < 10, 10–19, 20–29, 30–39, 40–49, 50–59, ≥ 60. Districts of cholera diagnosis were confined to Nkhatabay, Lilongwe, Mangochi, Blantyre. The Eligibility criteria for cases and controls were similar.

### Statistical analysis

All analyses were performed using R (version 4.3.3) [13] with relevant statistical packages, including tidyverse, gtsummary, and survival. Descriptive analyses were performed to characterize patient-level factors in fatal cholera patients and univariate and multivariate analysis were performed to investigate the association between patient-level factors and cholera mortality.

Prior to analysis, the dataset was cleaned to remove unimportant columns, address missing data, and reformat variables into appropriate categories or binary formats. Cases, defined as deceased patients, were matched to controls (defined as survivors) based on age-range and district of diagnosis. This matching process ensured comparability between groups, with the final dataset consisting of 87 matched cases and 87 controls.

In the descriptive analysis, frequencies and percentages were calculated for categorical variables and Chi-square tests applied to assess associations between these variables at a threshold of p < 0.05. Summary tables were generated to depict distributions of key characteristics, such as age, hydration status, and fluid management. These analyses provided a foundational understanding of the factors prevalent in fatal cholera cases.

In the univariate analysis, individual conditional logistic regression models were developed to identify associations between patient-level factors and mortality outcomes, accounting for the matched study design. A total of 10 univariate models were developed (each developed for Sex, Marital status, vaccine status, time to presentation, clinical status on admission, HIV status hydration status, fluid management, antibiotic treatment, and time in ward. All predictors with p-values of <0.05 were considered significant. A binary outcome variable distinguished survival status (alive = 0, deceased = 1).

For the multivariate analysis (**Table 1**), all significant variables from the univariate analysis (meeting the p < 0.2 threshold) were included in the multivariate conditional logistics regression model. Multicollinearity among predictors was assessed using the Variance Inflation Factor (VIF), and no variables demonstrated a high collinearity (with VIF > 10). Odds ratios (ORs) with 95% confidence intervals (CIs) were calculated for all predictors in the final model, and statistical significance was set at p < 0.05.

**Table 1:** Specification of the multivariate conditional logistic regression model used to assess patient-level risk factors associated with cholera mortality in Malawi. The table outlines the objective, outcome of interest, matching factors and primary exposures.

Objective	Type of model	Outcome of interest	Matching factors	Primary exposures
To identify patient-level factors associated with cholera mortality	Conditional logistic regression (matched case-control design)	Mortality status (0 = Alive, 1 = Deceased)	-Age group (<10, 10–19, 20–29, 30–39, 40–49, 50–50, > 60) -District of diagnosis (Nkhatabay, Lilongwe, Mangochi, Blantyre)	-Clinical status on admission (critical, stable) -Hydration status on admission (no dehydration, some dehydration, severe dehydration, unknown) - Adequacy of fluid management (adequate, inadequate) -Time spent in ward (<6 hr, > 6–24hr, > 24–48 hr, > 48hr-7dy, > 7dys)

Finally, the **S**trengthening the Reporting of Observational studies in Epidemiology (STROBE) reporting guidelines for case-control studies was used when writing this report [14].

### Ethical considerations

The study adhered to rigorous ethical standards to ensure the privacy and confidentiality of patient information. All data collected were anonymized, with identifiers removed to protect the identity of the individuals whose medical records were reviewed. As the study was a retrospective review of medical records, obtaining direct patient consent was not necessary. Nonetheless, institutional approvals were secured through support letters from the respective Research Coordinating Committees of the participating institutions. Furthermore, ethical approval (Protocol #23/04/4042) for the study was granted by the National Health Sciences Research Committee (NHSRC) in Malawi, ensuring compliance with national ethical guidelines for research.

## Results

### Baseline characteristics

The study analyzed 174 eligible cholera patient files, consisting of 87 identified cases matched to corresponding controls. **Table 2** summarizes the baseline characteristics of cases and controls. Among the enrolled patients, the largest proportion, comprising 20% (17/87) in cases and 21% (18/87) in controls, were children under 10 years old. A majority of patients across both groups were male, making up 55% (48/87) in cases and 52% (45/87) in controls. Additionally, most patients were single, with 46% (40/87) of cases and controls reporting single marital status.

**Table 2:** Baseline characteristics of enrolled cholera patients stratified by outcome status (cases and controls). Categorical variables are presented as counts and percentages [n(%)], and comparisons between groups using Pearson’s Chi-squared test. No statistically significant differences were observed between cases and controls across the baseline variables.

Variable	N	case, N = 87^1^	control, N = 87^1^	p-value^2^
**Age range of Participant (years)**	174			>0.9
< 10		17 (20%)	18 (21%)	
10-19		16 (18%)	15 (17%)	
20-29		16 (18%)	15 (17%)	
30-39		14 (16%)	14 (16%)	
40-49		7 (8.0%)	8 (9.2%)	
50-59		6 (6.9%)	6 (6.9%)	
> 60		11 (13%)	11 (13%)	
**Gender status**	174			0.3
Female		37 (43%)	42 (48%)	
Male		48 (55%)	45 (52%)	
Unknown		2 (2.3%)	0 (0%)	
**Marital Status**	174			>0.9
Single		40 (46%)	40 (46%)	
Married		38 (44%)	37 (43%)	
Unknown		9 (10%)	10 (11%)	
**District**	174			0.070
Central(Lilongwe)		3 (3.4%)	13 (15%)	
Northen(Nkhatabay)		13 (15%)	10 (11%)	
Southern (Blantyre)		8 (9.2%)	8 (9.2%)	
Southern(Mangochi)		63 (72%)	56 (64%)	

^1^n (%)

^2^Pearson’s Chi-squared test

Regarding the geographical distribution, most enrolled patients originated from Mangochi District, accounting for 72% (63/87) in cases and 64% (56/87) in controls. No statistically significant differences were identified in the proportions between age range, gender status, marital status, or district between cases and controls (p-values > 0.05).

### Patient-level factors in cholera patients

#### Time to hospital presentation after symptom onset.

**Table 3** shows the proportion of cholera patients who presented to the hospital at varying intervals after symptom onset, stratified by survival outcome. Among cases, a larger proportion (52%, 45/87) presented within the first 24–48 hours of symptom onset compared to other time intervals. Similarly, most controls (52%, 45/87) also presented between 24–48 hours of symptom onset. More cases were observed with a slightly higher percentage of patients - 23% (20/87) versus 16% (14/87) in controls- presenting after 48 hours of cholera symptoms. However, no statistically significant differences were identified in the proportions of time to presentation between cases and controls (p-value = 0.3).

**Table 3:** Comparison of clinical characteristics, vaccination status, and initial treatment factors among cholera patients characterised as cases and controls. All variables were assessed using Pearson’s Chi-squared test to evaluate differences between groups.

Variable	N	case N = 87^1^	control N = 87^1^	p-value^2^
**Time to presentation from symptom onset**	174			0.6
< 6 Hrs		6 (6.9%)	6 (6.9%)	
> 6–24 Hrs		16 (18%)	22 (25%)	
> 24–48 Hrs		45 (52%)	45 (52%)	
> 48 Hrs		20 (23%)	14 (16%)	
**Vaccine status**	174			0.7
Unknown		24 (28%)	27 (31%)	
Unvaccinated		63 (72%)	60 (69%)	
Vaccinated		0 (0%)	0 (0%)	
**HIV status**	174			0.3
Negative		4 (4.6%)	2 (2.3%)	
Positive		2 (2.3%)	0 (0%)	
Unknown		81 (93%)	85 (98%)	
**Clinical status on admission**	174			0.11
Critical		64 (74%)	53 (61%)	
Stable		23 (26%)	34 (39%)	
**Hydration status on admission**	174			0.10
No dehydration		1 (1.1%)	4 (4.6%)	
Some Dehydration		35 (40%)	42 (48%)	
Severe Dehydration		50 (57%)	37 (43%)	
Unknown		1 (1.1%)	4 (4.6%)	
**Fluid management (first 2–6 hours)**	174			<0.001
Adequate MGT		45 (52%)	84 (97%)	
Inadequate MGT		42 (48%)	3 (3.4%)	
**Antibiotic management**	174			0.7
Given (doxycycline/ azithromycin/ciprofloxacin)		82 (94%)	84 (97%)	
Not Given		5 (5.7%)	3 (3.4%)	
**Time spent in ward**	174			0.3
6–24 Hrs		12 (14%)	5 (5.7%)	
> 24–48 Hrs		27 (31%)	25 (29%)	
> 48 Hrs-7 Days		34 (39%)	39 (45%)	
> 7 Days		14 (16%)	18 (21%)	

^1^n (%).

^2^Pearson’s Chi-squared test.

#### Cholera vaccination and HIV status.

The cholera vaccination status did not differ significantly between cases and controls (p = 0.7) (**Table 3**). Most cases (72%, 63/87) enrolled in this study were not vaccinated with the oral cholera vaccine and 28% (24/87) had an unknown vaccination status. This was a similar observation in controls, where 69% (60/87) had not received the oral cholera vaccine and 31% (27/87) had an unknown vaccination status.

Regarding the HIV status of enrolled patients, most cases and controls- 93% (81/87) and 98% (85/87) respectively- had an unknown status.

#### Clinical status on admission.

Among the 174 patients included in the study, clinical status on admission did not differ significantly between cases and controls (p = 0.11) (**Table 3**). Although a higher proportion of cases (74%, 64/87) were admitted in critical condition compared to controls (61%, 53/87), this difference was not statistically significant. Similarly, 26% (23/87) of cases were admitted in stable condition, compared to 39% (34/87) of controls.

#### Reported hydration status on admission.

**Table 3** illustrates the proportions for hydration status of cholera patients upon hospital admission, categorized as either “no dehydration”, “some dehydration” and “severe dehydration” and stratified by survival outcome. Most (57%, 50/87) cases were admitted with severe dehydration while 40% (35/87) had some dehydration. Conversely, for controls, there were more (48%, 42/87) patients with some dehydration than severe dehydration (43%, 37/87). However, these differences, between cases and controls, were not statistically significant (p-value = 0.10).

#### Adequacy of fluid management.

**Table 3** highlights the adequacy of intravenous fluid management among cholera patients, categorized as either adequately or inadequately managed based on whether the type, volume and timing of intravenous fluids administered in the first 6 hours aligned with the standard cholera treatment guidelines for managing dehydration. Among the cases, 52% (45/87) were adequately managed with IV fluids while 48% (42/87) were classified as inadequately managed. In comparison, the controls had more (97%, 84/87) patients adequately managed with IV fluids than those inadequately managed. These differences in the proportions between cases and controls were statistically significant (p < 0.001).

#### Antibiotic management.

There was no statistically significant difference in antibiotic administration between cases and controls (p = 0.70). Almost all patients in both groups received antibiotics during their admission, with 94% (82/87) of cases and 97% (84/87) of controls prescribed either doxycycline, azithromycin, or ciprofloxacin. A small proportion did not receive antibiotics: 5.7% (5/87) of cases and 3.4% (3/87) of controls.

### Time spent in the hospital ward

**Table 3** presents the distribution of total time spent in the hospital ward by cholera patients, categorized into four time intervals (6–24 hours, 24–48 hours, 48 hours–7 days, and >7 days) and stratified by survival outcome. The distribution of time spent in the ward was similar across both groups, with most patients staying more than 24 hours. Specifically, 39% (34/87) of cases and 45% (39/87) of controls stayed between 48 hours and 7 days, while 16% (14/87) of cases and 21% (18/87) of controls remained hospitalized for more than 7 days. These differences in the proportions observed between cases and controls were not statistically significant (p-value = 0.15).

### Risk factor analysis for cholera mortality

In the univariate conditional logistic regression analysis, four variables were significantly associated with cholera mortality out of the ten tested (**Table 4**). Clinical status on admission, based on initial vital sign assessment, was significantly associated with mortality: patients admitted in a clinically stable condition had 56% reduced odds of death compared to those admitted in critical condition (OR = 0.44, 95% CI: 0.22–0.88, p = 0.021). Hydration status on admission was also significantly associated with mortality overall (p = 0.01). However, the specific category of severe dehydration did not reach statistical significance when compared to the reference group (no dehydration), despite showing a trend toward increased risk (OR = 6.40, 95% CI: 0.66–62.0, p = 0.11). Adequacy of fluid management, defined by whether the timing and volume of fluid administration during the initial 6 hours aligned with national treatment guidelines, was a strong predictor of mortality. Patients who received inadequate fluid management had significantly increased odds of death (OR = 39.71, 95% CI: 8.98–175.5, p < 0.001). Finally, time spent in the ward was not statistically significant overall (p = 0.09), but patients who remained hospitalized for more than 7 days had significantly lower odds of death compared to those who stayed for less than 24 hours (OR = 0.15, 95% CI: 0.03–0.83, p = 0.03). These variables were included in the multivariable regression analysis. Conversely, other variables such as sex, marital status, HIV status, vaccine status, time to presentation, HIV status, and antibiotic treatment did not demonstrate statistically significant associations with mortality (p > 0.05).

**Table 4:** Univariate conditional logistic regression results outlining significant predictors of cholera mortality. Variables were individually tested using conditional logistic regression, adjusting for age group and district via stratification. P-values indicate the strength of association between each variable and mortality.

Variable	P- value	Include in the multivariable model? (at p < 0.2 significance level)
Sex	0.20	No
Marital status	0.70	No
Vaccine status	0.40	No
Time to presentation	0.40	No
Clinical status on admission	**0.021**	Yes
HIV status	0.30	No
Hydration status	**0.01**	Yes
Fluid management	**<0.001**	Yes
Antibiotic treatment	0.30	No
Time in ward (>7days)	**0.03**	Yes

In the multivariable conditional logistic regression model (**Table 5**), which included 174 matched observations and adjusted for age group and district, inadequate fluid management was the only factor significantly associated with increased odds of death. Patients who received inadequate fluid resuscitation in the first 6 hours of admission had 45 times higher odds of dying compared to those who received adequate fluid management (adjusted OR = 45.26, 95% CI: 9.09–225.3, p < 0.001). Other variables, including clinical status on admission, hydration status, and time spent in the ward, were not statistically significant in the adjusted model (p > 0.05).

**Table 5:** Multivariable conditional logistic regression model of patient-level factors associated with cholera mortality in Malawi. The model includes 174 matched observations, with matching on age group and district. All participants were assumed to have equal risk time, consistent with the matched case-control design. Adjusted Odds ratios, 95% confidence intervals, and p-values are presented for each predictor. Nagelkerke’s R² indicates the overall explanatory power of the model.

		Cholera mortality		
*Predictors*	*Reference*	*Adjusted OR*	*CI*	*P-value*
Clinical status on admission [Stable]	[Critical]	0.56	0.19 – 1.64	0.293
Hydration status [Some Dehydration]	[No dehydration]	9.32	0.42 – 206.17	0.158
Hydration status [Severe Dehydration]	[No dehydration]	6.12	0.26 – 144.61	0.262
Hydration status [Unknown]	[No dehydration]	4.11	0.09 – 196.98	0.474
Fluid management [Inadequate MGT]	[Adequate MGT]	45.26	9.09 – 225.30	**<0.001**
Time in ward>24–48 Hrs	[<24 Hrs]	0.34	0.06 – 2.08	0.244
Time in ward>48 Hrs-7 Days	[<24 Hrs]	0.22	0.04 – 1.26	0.089
Time in ward [>7 Days]	[<24 Hrs]	0.24	0.04 – 1.57	0.137
Observations		174		
R^2^ Nagelkerke		0.461		

## Discussion

This case-control study identifies one key risk factor for mortality among cholera patients admitted in Malawi during the 2022–2023 cholera outbreak in high-burden districts. We reveal that inadequate intravenous fluid management in the first 6 hours is strongly associated with fatal outcomes, significantly increasing the odds of mortality by over 45-fold compared to patients who received adequate IV fluid management. In this study, inadequate fluid management was defined as the failure to administer the appropriate type, volume, and timing of intravenous fluids in the first 6 hours of admission, based on the patient’s initial hydration status and in accordance with national cholera treatment guidelines. This underscores the critical role of timely and appropriate fluid resuscitation in the management of cholera, particularly for patients presenting with dehydration.

Among the fatal cholera cases in this study, 48% were classified as having received inadequate intravenous fluid management, highlighting a significant gap in adherence to standard treatment guidelines. Cholera causes massive fluid and electrolyte loss due to the action of cholera toxin on the intestinal mucosa, leading to hypovolemic shock, metabolic acidosis, and, if untreated, rapid death [15]. These findings reinforce the importance of appropriate and timely fluid resuscitation in managing cholera patients, particularly those presenting with signs of dehydration. According to the World Health Organization’s recommended treatment for severe cholera, patients require rapid IV fluid rehydration within the first 2–6 hours, followed by antibiotics and Oral Rehydration Salts (ORS) over the next two days [8]. On average, severe cases necessitate approximately 7 litres of intravenous fluids and 14 litres of ORS over the course of treatment [15]. Given these significant fluid requirements, any deviation or delay in administering adequate rehydration therapy can have severe and often fatal consequences, emphasizing the pivotal role of strict adherence to fluid management protocols in reducing mortality among cholera patients.

Our findings on the strong association between inadequate IV fluid management and cholera mortality are consistent with existing literature [16]. For instance, during one of the deadliest cholera outbreaks in the Democratic Republic of Congo in 1994, where an estimated 12,000 refugees died, inadequate fluid management was attributed to delayed IV fluid administration and incorrect dosing that deviated from standard treatment protocols [16]. These shortcomings were largely driven by shortages of IV fluids and clean water, the latter limiting the availability of Oral Rehydration Salts [16]. Similarly, in our study, the high proportion of inadequate IV fluid administration among fatal cases may reflect broader resource limitations in cholera treatment units, as previously documented in national health system assessments [4,5]. This situation was likely exacerbated by the infrastructural damage caused by Tropical Cyclone Freddy, which had already been reported to impair health facility operations and supply chains [3–5]. The cyclone also contributed to population displacement and overcrowding in treatment units, potentially overwhelming the healthcare system’s capacity to deliver adequate care. These conditions may have hindered the consistent delivery of IV fluids, making it difficult to meet the recommended average of 7 litres per patient within the critical first 2–6 hours of care. To address such challenges, we emphasize the importance of strengthening cholera surveillance systems in Malawi by integrating meteorological data to predict climate-related influences on health and improve preparedness for future outbreaks.

The study further demonstrated the potential influence that hydration status on admission can have on patient outcomes. We observed that patients presenting with severe dehydration were overrepresented among fatal cases, indicating that delays in initial resuscitation and fluid replacement likely contributed to the observed mortality. While severe dehydration on admission did not yield statistically significant associations with mortality in this study, the trend observed aligns with existing evidence from cholera outbreaks in other African countries, where severe dehydration, alongside older age, gender and residential location, are key predictors of poor outcomes [17]. Therefore, early identification and aggressive management of severe dehydration in cholera patients remain pivotal in reducing fatalities.

Time to hospital presentation was also assessed as a potential factor influencing mortality. Although the findings in this study were not statistically significant, the time patients present to hospital after symptoms develop is an important factor to discuss, especially because most cholera patients in this study (cases and controls) presented to hospital after 24 hours. This delay likely reflects the existing barriers to healthcare access in Malawi, as highlighted by Msokwa [18], emphasising geographic challenges, financial constraints, and infrastructural limitations. Such barriers can exacerbate cholera outcomes during outbreaks. Consistent with this observation, Kelly Osezele Elimian, Anwar Musah [19] demonstrated that a delay of more than two days after the onset of cholera symptoms significantly increased the odds of cholera-related death (aOR 2.08, 95% CI: 1.52–2.85). We reinforce the critical need for strengthening community-based awareness campaigns and implementing robust early referral systems to ensure timely access to care, thereby improving patient outcomes during cholera outbreaks.

While this study provides valuable insights into the factors associated with cholera mortality in Malawi, several limitations must be acknowledged. The retrospective design and reliance on medical record reviews may have introduced information bias, particularly in the categorization of fluid management adequacy. This categorization depended on the quality of documentation, and any errors or inconsistencies in recording the volume, type, and timing of IV fluids administered could have resulted in the misclassification of patients. Such misclassification may have distorted the true relationship between fluid management and mortality. However, this limitation was mitigated by employing trained research Medical Officers to lead data collection teams, ensuring that patients were carefully categorized as “adequately” or “inadequately” managed with IV fluids based on thorough reviews of patient files.

Additionally, the lack of granular data on key patient-level factors, such as health-seeking behaviors, may have reduced the statistical power to detect significant associations for some predictors. The absence of detailed data on health-seeking behaviors, for instance, limits the ability to identify root causes of delayed hospital presentations and broader social or economic barriers to healthcare access. Furthermore, variables such as cholera vaccination status and HIV status had substantial missing data, attributed to incomplete patient file documentation, this undoubtedly limited our attempt to correctly evaluate their association to mortality. Similarly, the absence of documentation on oral rehydration therapy intake in patient files precluded its inclusion in our analyses. Taken together, these factors compromise the generalisability of our findings to all Malawian treatment units.

Finally, another limitation of this study is the 1:1 matching ratio between cases and controls. Employing a higher matching ratio—such as 1:3 or 1:5—could have increased the statistical power and precision of our estimates. However, resource and time constraints limited our ability to review a larger number of medical records, as the study was conducted in the context of an active cholera outbreak and aimed to provide timely data to inform the national response.

Despite these limitations, the findings highlight critical gaps in cholera treatment and provide a basis for targeted interventions. Future studies should aim to address these challenges by employing prospective designs and utilizing comprehensive data collection tools to minimize information bias and capture detailed patient-level factors. These efforts will improve the ability to identify actionable strategies for reducing cholera mortality.

## Conclusion

This study identified inadequate intravenous fluid management within the first 6 hours of admission as a key risk factor for cholera mortality in Malawi’s treatment units during the 2022–2023 outbreak. The findings underscore the critical importance of timely and appropriate fluid resuscitation in accordance with treatment guidelines to prevent fatal outcomes.
